# Fabrication of Spiral Low-Cost Microchannel with Trapezoidal Cross Section for Cell Separation Using a Grayscale Approach

**DOI:** 10.3390/mi14071340

**Published:** 2023-06-30

**Authors:** Mohamed Adel, Ahmed Allam, Ashraf E. Sayour, Hani F. Ragai, Shinjiro Umezu, Ahmed M. R. Fath El-Bab

**Affiliations:** 1 Department of Mechatronics and Robotics Engineering, Egypt-Japan University of Science and Technology (E-JUST), Alexandria 21934, Egypt; ahmed.rashad@ejust.edu.eg; 2 Mechanical Engineering Department, Helwan University, Cairo 11792, Egypt; 3 Department of Electronics and Communications Engineering, Egypt-Japan University of Science and Technology (E-JUST), Alexandria 21934, Egypt; ahmed.allam@ejust.edu.eg; 4 Molecular Biomimetics Research Group, Animal Health Research Institute, Agricultural Research Center, Giza 12618, Egypt; sayourashraf@gmail.com; 5 Electronics and Communications Department, Faculty of Engineering, Ain Shams University, Cairo 11517, Egypt; hani_ragaai@eng.asu.edu.eg; 6 Department of Modern Mechanical Engineering, Waseda University, 3-4-1 Okubo, Shinjuku-ku, Tokyo 169-8555, Japan; umeshin@waseda.jp

**Keywords:** spiral microchannel, grayscale, CO_2_ laser, cell separation

## Abstract

Trapezoidal cross-sectional spiral microfluidic channels showed high resolution and throughput in cell separation in bio-applications. The main challenges are the complexity and high cost of the fabrication process of trapezoidal cross-sectional channels on the micro-scale. In this work, we present the application of grayscale in microfluidic channel design to overcome the complexity of the fabrication process. We also use direct engraving with a CO_2_ laser beam on polymethyl methacrylate (PMMA) material to drastically reduce the microfluidic chip’s cost (to <30 cents) and fabrication time (to 20 min). The capability of the present fabrication methodology for cell sorting applications is demonstrated through experimental tests for the separation of white blood cells (WBCs) from whole blood at different dilution factors. The experimental results indicated that an 800 µL/min flow rate provided the optimal separation efficiency using the fabricated chip. A 90.14% separation efficiency at 1% hematocrit diluted blood sample was reported.

## 1. Introduction

Separating specific cells or particles from a background mixture is a critical process in many bio-applications, and this step usually comes before other processing stages [1]. An important example is the separation process of white blood cells (WBCs) from whole blood. It is noteworthy that blood is composed of liquid plasma, cells, and platelets. Blood cells are either red cells (erythrocytes) or white cells (leucocytes). Human red blood cells (RBCs) have a biconcave shape with a diameter of about 7–8 µm [2]. The average diameter of WBCs is 12–15 µm [3]. Blood platelets are circulating nuclear fragments of 3 to 4 µm in diameter [4].

The isolated WBCs are used for disease diagnostics and provide valuable information about the immune system. Centrifugation and flow cytometry are two examples of traditional methods for cell sorting processes [5,6]. In addition to the expensive time-consuming instruments and the need for skilled personnel, the traditional methods cannot be integrated with on-chip systems. In recent years, high throughput microfluidic cell sorting chips with different designs have been developed to provide the ability for integration into point-of-care systems [7]. Cell sorting techniques in microfluidic chips are classified into active and passive methods [8,9]. These methods are based on the unique characteristics of cells or particles [1].

Active sorting methods, such as dielectrophoresis (DEP) [10], magnetophoresis [11], and acoustophoresis [12], utilize externally applied forces to separate cells. Conversely, there is no need for external forces for cell separation in passive methods. In contrast to active methods, passive methods are widely spread due to their low fabrication cost, high throughput, and simple channel geometries [7,13].

Inertial microfluidics is a passive separation technique that addresses the trade-off between efficiency and throughput compared to other passive techniques, such as pinched-flow fractionation (PFF) [14] and hydrodynamic filtration [15]. Techniques of cell sorting using inertial microfluidics depend on the hydrodynamic forces acting on cells [7]. Based on their size, particles inside the inertial microfluidic channels are forced to migrate in different equilibrium positions, allowing a continuous label-free particle separation process.

Despite its simplicity and ease of fabrication, straight-channel inertial microfluidics does not provide enough force for separation in many applications [16]. Therefore, spiral channel inertial microfluidics are widely used for particle or cell separation. In a spiral microfluidic chip, based on the channel dimensions, larger particles migrate toward the inner wall of the channel, and in comparison, smaller particles focus near the outer wall [8,17]. In addition to blood cell separation, spiral microfluidics are used in other biological applications, such as circulating tumor cell (CTC) separation [18] and sperm cell separation [19].

Rectangular cross-sectional spiral microfluidic channels have been used in different biological particle separation applications [7,18,19,20,21,22]. However, their use in applications like WBCs separation from whole blood, which require higher forces for focusing cells on different streamlines, needs to be more efficient. This lack in efficiency happens because of the large number of RBCs in the blood sample, leading to cell-to-cell interactions that overcome the hydrodynamic forces at the working flow rates.

Guan et al. [23] proposed trapezoidal cross-sectional spiral microfluidic channels to generate a higher force and produce higher separation resolution. Further research was then achieved using trapezoidal cross-sectional channels in particle separation. The separation of Leukocytes from blood samples [24] and the isolation of CTC [25] were performed using trapezoidal cross-sectional channels. The enhancement of separation resolution and efficiency (>80%) was demonstrated using trapezoidal cross-sectional channels. Akbarnataj et al. [26] proposed a PDMS microchannel with a trapezoidal cross section and elliptic spiral configuration to separate CTCs from blood samples. CTC purity values ranging from 88% to 90% have been achieved through experimental tests. However, the complexity of fabrication and high cost are still the main challenges. The complexity of the fabrication of trapezoidal cross-sectional channels encouraged researchers to propose the application of channels with different cross-sections rather than trapezoidal ones. A u-shaped cross-sectional spiral channel for WBCs separation from whole blood was proposed by Mehran et al. [13].

The standard soft lithography methodology has been used to fabricate microfluidic systems in the last two decades. However, the need for a clean room environment and expensive equipment, such as photolithography machines and photomasks, increases the fabrication cost and time [27,28,29]. Despite its suitability for the fabrication of microfluidic chips due to its nontoxicity, biocompatibility, and good thermal stability, PDMS has some drawbacks [1]. In addition to its mechanical softness and its hydrophobicity, the most serious drawback of PDMS is that it can withstand a specific range of pressure [30,31]. High pressures cause elastic deformation in the channel resulting in differences between the expected and the real flow rates inside the microfluidic chip [31].

To avoid these drawbacks, direct writing on a glass wafer using a femtosecond laser for the fabrication of trapezoidal cross-section channels was proposed [1,32]. Due to the high power required for glass machining, the process is performed using multi-layer fabrication. The laser ablation step is followed by glass etching and multiple cleaning steps using a specific chemical cleaning machine. The machined wafer is then bonded to another cover wafer thermally at 620 °C. The microfluidic chip is then diced from the whole wafer. In conclusion, the proposed method requires specific machines that increase the fabrication cost and is performed in long-time multi-stages. Zhu et al. [33] introduced a size-based cell sorter with trapezoidal cross-section spiral channels by assembling two pieces of silicon rubber sheet plates of varying thicknesses. The sheets are patterned using an ultraviolet (UV) laser cutting and then bonded with upper and lower covers through plasma-activated bonding. The proposed method demonstrated high efficiency in separating tumor cells from blood samples. However, like PDMS, plasma-bonded microfluidic chips have a relatively narrow working pressure range.

Due to its availability, low cost, biocompatibility, and transparency, polymethyl methacrylate (PMMA) material is used to fabricate microfluidic channels in different bio-applications, such as DNA and PCR chips [34,35]. Direct engraving of microfluidic systems in different materials using a laser beam has been used in different bio-applications [36,37]. The use of CO_2_ laser for micromachining on PMMA material was studied by many researchers [38,39,40,41,42]. The fabricated channels using CO_2_ laser in the literature are limited to Gaussian cross-sectional shape due to the Gaussian nature of laser beam [38] or rectangular cross-sectional shape [42].

Grayscale in microfabrication refers to a technique where varying levels of gray shades are used to create three-dimensional (3D) structures with height variations on a substrate [43]. Laser lithography is the most commonly used method for grayscale patterning, where a UV laser beam is used to pattern a photosensitive material (photoresist) coated on the surface of the substrate. This substrate is then subjected to further processes such as deposition or etching. For instance, Youn et al. [44] presented a method for using a positive photoresist to produce layers with different thicknesses as a function of grayscale levels (ranging from 0 to 99) using a UV laser beam, then using reactive ion etching (RIE) to fabricate a quartz multitier mold. This fabrication technique is time-consuming and requires a cleanroom environment and expensive equipment.

Li et al. [45] presented a grayscale surface patterning method for mask-free fabrication using a femtosecond laser based on spatiotemporal interference. The proposed method achieved the desired pattern by employing a spatial light modulator capable of controlling the distribution of phase differences of laser beams. In this work, we present the application of grayscale design to overcome the complexity of the fabrication process of trapezoidal cross-sectional spiral channels. The grayscale design of the channel is applied to the PMMA material through direct writing with a CO_2_ laser beam without the need for additional modular components. This fabrication technique eliminates the need for a photomask and a clean room environment, reducing the fabrication time and the cost of the microfluidic chip.

This work presents a simple fabrication technique of microfluidic channels with trapezoidal cross section for bio-applications using a grayscale drawing approach in channels’ design. Using CO_2_ laser and PMMA material reduces the fabrication time and the microfluidic chip cost and allows the application of fluids with high working flow rates to the chip. The capability of the presented fabrication technique to be applied in microfluidic applications is demonstrated by testing on WBCs separation from whole blood. The present article is organized as follows. The used material and applied methods for microfluidic chip fabrication and testing are presented in Section 2. Then, Section 3 reveals and discusses the results of the performed experimental work for the chip fabrication and the WBCs separation test. Finally, conclusions are reported in Section 4.

## 2. Materials and Methods

### 2.1. Spiral Channel Operational Concept

In a bounded spiral microfluidic channel, the flow velocity mismatch between the fluid in the center of the channel and near the channel walls causes a secondary flow [16]. Due to the centrifugal acceleration, resulting from the curvilinear nature of spiral channels, particles at the center of the channel have more considerable inertia than others. This difference in the inertia of fluid elements creates a pressure gradient across the channel. As a result, fluid near the walls re-circulates inward, forming two circulating vortices [46]. Therefore, particles in the flow experience inertial lift forces and secondary Dean drag forces. The secondary Dean drag forces entrain particles within streamlines of vortices, and inertial lift forces tend to hold particles at specific equilibrium positions [16]. The magnitude of the inertial lift force and Dean drag force determines the final equilibrium position of each particle.

**Figure 1:**
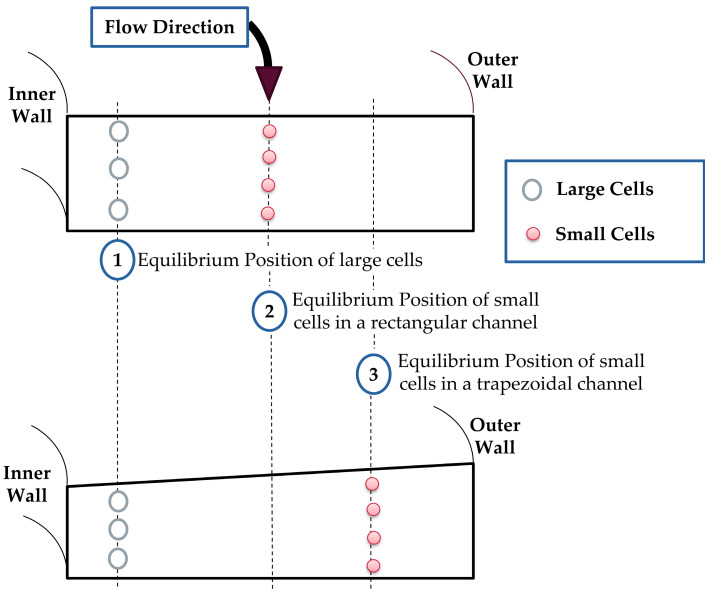
Schematic diagram of the equilibrium positions of cells in rectangular and trapezoidal cross-section spiral microfluidic channels.

The ratio of the inertial lift force to the Dean drag force is proportional to a^3^, where a is the particle diameter [13]. Therefore, larger particles migrate toward the inner wall of the channel in a specific equilibrium position, and smaller particles focus on a different equilibrium position. In rectangular cross-section spiral channels, Dean cores of vortices are approximately centered between the inner and outer walls. While in trapezoidal channels, they are shifted toward the outer wall of the channel [23]. Therefore, the distance between the equilibrium positions of the large and small cells increases. As a result, the separation efficiency enhances. Figure 1 shows a schematic diagram of the equilibrium positions of cells, based on their size, in rectangular and trapezoidal cross-section spiral microfluidic channels.

### 2.2. Concept of Fabrication Using Grayscale Patterning

The laser power level and scanning speed are the two main parameters to control the depth of a specific pattern on the machined material when using the laser direct writing fabrication method. For microfluidic channel fabrication, the depth of the channel increases as the laser power level increases. Conversely, increasing the laser scanning speed reduces the depth of the channel. Therefore, different combinations of laser power and speed values can be used to obtain the required depth. The whole design is performed with a constant depth in each machine run because the power and speed are set once before machining. To solve this problem, we propose using grayscale drawing to obtain more than one depth in the same machine run. The laser power and speed are set constants using this approach, and the RGB value of each color controls the depth.

**Figure 2:**
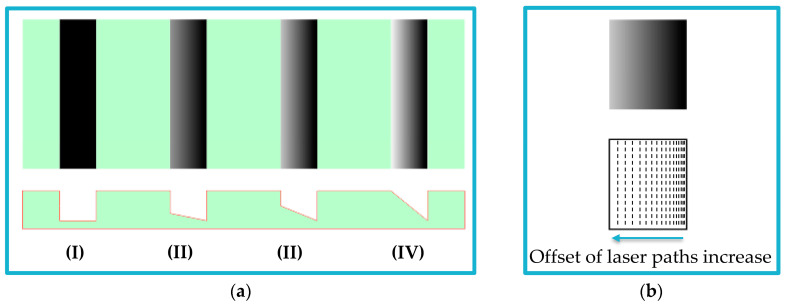
Schematic diagram of applying grayscale concept for microfluidic channels fabrication: (**a**) Grayscale channel drawing (upper) and expected channel cross-section (lower). Uniform fill is applied for rectangular cross-section channels (I). Gradual fill is used for trapezoidal cross-section channels: (II) black to 40% black, (III) black to 20% black, and (IV) black to white. (**b**) Concentrations of laser paths based on the color RGB value. High concentration of laser paths at low RGB values, and a lower concentration at high RGB values.

In grayscale, the RGB value varies from 0 (black) to 255 (white). A uniform fill with a constant RGB value is used for rectangular cross-section channel fabrication in microfluidic applications. While for the trapezoidal cross-section channel, a gradual fill, starting with a specific RGB value and ending with another value, is applied. The trapezoidal slope can be controlled by the starting and ending RGB values. Figure 2a illustrates the concept of applying the grayscale approach for microfluidic channel fabrication and the effect of changing the color RGB value. The offset value between laser paths is constant throughout the process in uniform fill. On the other hand, the offset between laser paths changes based on the color RGB value in gradual fill. Therefore, the laser ablation concentration is higher in dark areas than in lighter ones (see Figure 2b).

### 2.3. Microfluidic Chip Design and Fabrication

**Figure 3:**
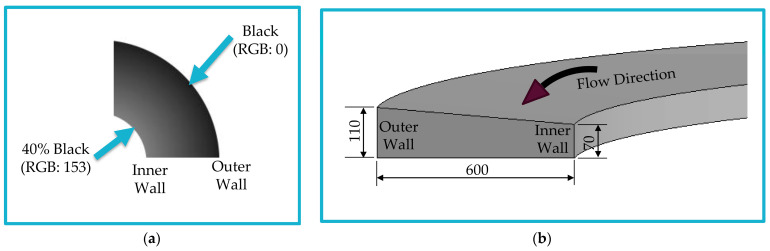
(**a**) A segment of the designed spiral channel filled with grayscale. (**b**) A 3D cross-section of the spiral microfluidic channel (The dimensions are in µm).

The microfluidic chip was designed using the grayscale approach discussed in Section 2.2. A spiral channel was drawn and filled with grayscale (see Figure 3a). The grayscale RGB values were selected based on the required channel depth and slope. The chip consists of an 8-loop spiral channel with a 7 mm initial diameter and a 1.5 mm loop-to-loop distance. The chip is designed to be single-input-double-output with a trapezoidal cross section of 600 µm width, 70 µm inner depth, and 110 µm outer depth. The 600 µm channel is split into two outlets (inner and outer) with a ratio of 1:2, respectively. The dimensions of the channel should be compatible with the size and deformability range of the target cells. These dimensions play a crucial role in determining the effectiveness and efficiency of the cell separation process within the chip. Therefore, the dimensions of the channel were carefully chosen by referring to the existing scientific literature [1,23,24,25] to ensure the effectiveness and efficiency of the chip in its intended applications. Figure 3b presents a schematic diagram of the designed spiral microfluidic channel cross-section.

**Figure 4:**
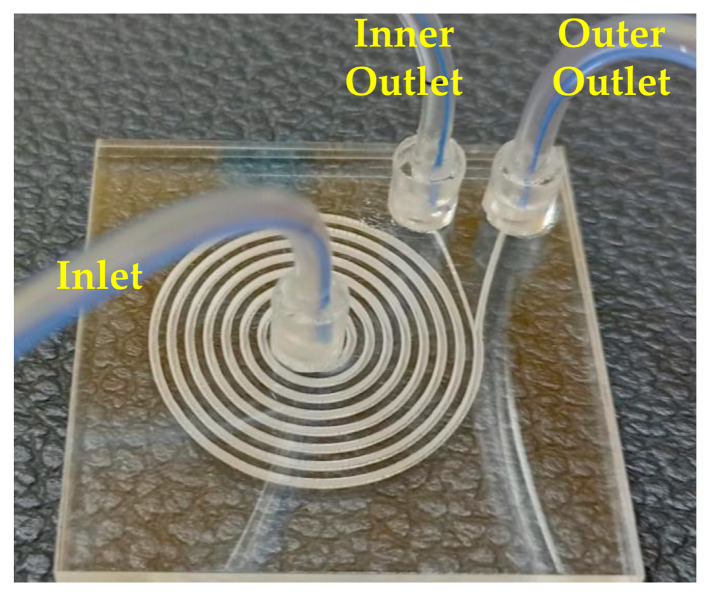
The trapezoidal cross-section spiral microfluidic chip fabricated using the proposed methodology in the current work.

The fabrication was performed using a CO_2_ laser machine (VLS3.5, Universal Laser Systems, Kanagawa, Japan) with a 30-watt laser tube. A laser lens (HPDFO, Universal Laser Systems, Japan) of 30 µm focal spot was used. The machine was first calibrated to determine the equivalent depth in PMMA to each RBG value of the grayscale drawing. The machine calibration step is carried out once for each used lens. PMMA sheets (Spiroplastic, Cairo, Egypt) of 2 mm thickness were used to fabricate the microfluidic chip in this work. The laser beam was applied with 1000 pulses per inch (PPI) to develop the microfluidic chip by patterning the PMMA sheets. The channels were engraved in the lower layer, while the inlet and the two outlets were drilled into the upper cover. The engraved surface was cleaned and thermally processed. The two layers were then bonded thermally in a natural ventilation lab oven (STF-N 80, FALC Instruments, Treviglio, Italy). After bonding, 1.8 mm outer diameter connection tubes (ULTRAMED, Assiut, Egypt) were fixed and glued in the inlet and outlet holes. Figure 4 shows the fabricated chip after bonding and fixing connection tubes.

After fabrication, the channel dimensions and the surface roughness of the machined surface were measured using a 3D laser microscope (KEYENCE VK-X100, Keyence Corporation of America, Itasca, IL, USA). We repeated this measurement process after subjecting the microfluidic chip to the same thermal conditions as the bonding process to test the dimensions’ stability. Deionized (DI) water was pumped to the microfluidic chip through the input port at different flow rates for leakage testing.

### 2.4. WBCs Separation from Whole Blood

WBCs separation from whole blood experiment was performed to verify the fabricated microfluidic chip’s ability to be used in cell sorting applications and potential isolation and detection of intracellular microorganisms like brucellae.

#### 2.4.1. Sample Preparation

Whole blood samples were freshly collected from a 31-year-old male in sterile blood collection tubes (VACO MED, Cairo, Egypt) with sodium citrate as an anticoagulant. Sodium citrate was chosen for being relatively less destructive to blood cells as compared to EDT [47]. A complete blood count (CBC) was performed, and 51% hematocrit was measured for the donor’s blood sample. The whole blood sample was diluted to reduce the cell-to-cell interaction due to the high concentration of RBCs in the blood. A sample tube was prepared by mixing the whole blood in DI water with a ratio of 1:51, respectively. Therefore, the resulting diluted sample was 1% hematocrit. To prepare 2% and 3% hematocrit blood samples, the whole blood was diluted in DI water with ratios of 1:25.5 and 1:17, respectively. Diluting using DI water causes lysis of RBCs. When the target is separation of RBCs, it is recommended to use saline solutions for dilution to avoid any lysis of RBCs.

#### 2.4.2. Experimental Separation Process

**Figure 5:**
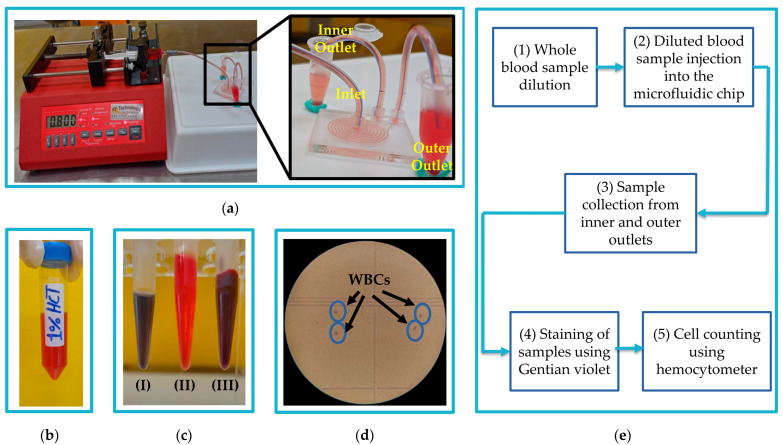
(**a**) The fabricated trapezoidal cross-section microfluidic chip setup for the white blood cells (WBCs) separation process. (**b**) A 1% hematocrit diluted blood sample. (**c**) Blood samples: (I) stained sample collected from the inner outlet of the chip, (II) sample collected from the outer outlet of the chip, and (III) whole blood sample. (**d**) Optical microscope hemocytometer image for WBCs counting. (**e**) The overall experimental process of WBCs separation and enumeration.

The diluted blood samples were pumped into the spiral microfluidic chip through the inlet port using 5 mL sterile syringes (Jiangxi Hongda Medical Equipment Group, Nanchang, China) fixed on a syringe pump (NE-4000, New Era Systems, Grant, FL, USA). A fresh sample was prepared just before injection to the chip to avoid the total lysis of RBCs in the injected sample and to ensure an accurate separation process. Samples were collected through the inner and outer outlets in 1.5 mL reaction tubes (Greiner Bio-One Gmbh, Frickenhausen, Germany). Figure 5a illustrates the chip setup for the WBCs separation process. Experiments were conducted at different flow rates to define the most optimal. Each experiment was repeated three times to reduce errors. After each experiment, a cleaning process was performed to remove any residues on the walls of the channel by pumping DI water at 3 mL/min to the chip.

The WBCs were counted in the samples collected from both outlets after a delay of 30 min to ease the counting process without requiring further dilutions. For WBCs visualization, the collected samples were stained using Gentian violet (Sigma-Aldrich, St. Louis, MO, USA). Figure 5b shows a diluted 1% hematocrit sample, and Figure 5c presents tubes of a whole blood sample, a collected sample, and a stained sample. Cell counting was performed using a hemocytometer (Paul Marienfeld GmbH & Co. KG, Lauda-Königshofen, Germany). An image of the hemocytometer with WBCs under a trinocular compound epi-fluorescence microscope (FM580TA, AmScope, Irvine, CA, USA) is shown in Figure 5d. The WBCs separation efficiency was calculated as the ratio of the number of WBCs in the inner outlet to the total number of WBCs in both outlets. In contrast, the WBCs recovery rate was calculated as the ratio of the number of WBCs in the outlet to the total number of WBCs in the inlet. The entire experimental WBCs separation process is described in Figure 5e.

## 3. Results and Discussion

### 3.1. Microfluidic Platform Fabrication

**Figure 6:**
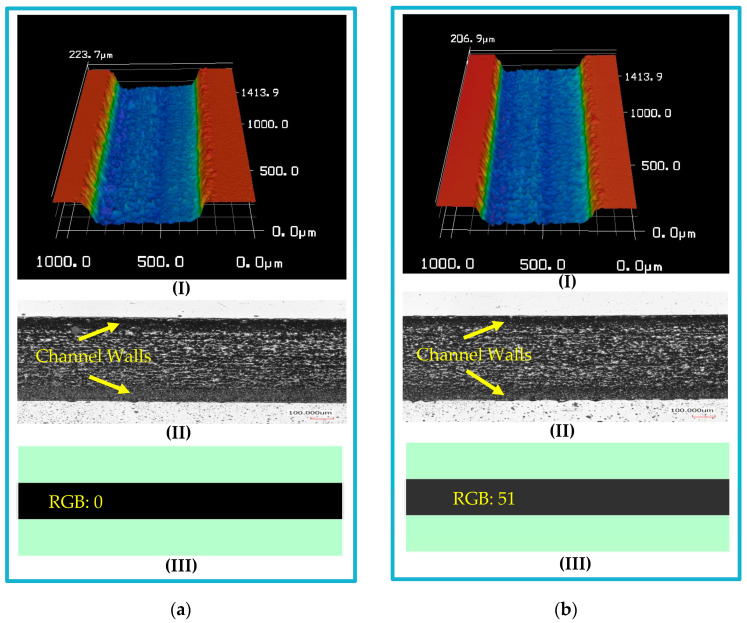
Microfluidic channels fabricated by direct writing of CO_2_ laser beam on polymethyl methacrylate (PMMA) applying the proposed grayscale approach: (I) 3D channel image, (II) optical channel image, and (III) the used color in the design of the channel. A uniform fill is applied in the channel design for rectangular cross-sections. Black (RGB: 0) is used for high-channel depth (**a**), and 80% black (RGB: 51) is used for a lower depth (**b**).

Uniform fills of different grayscale values were applied for rectangular cross-section channels of the same width to measure the depth for each corresponding RGB value. After direct writing of the CO_2_ laser beam on PMMA, a notable difference in depth was observed. Figure 6 presents microfluidic channels fabricated with operating conditions of 30 watt laser power, 250 mm/s scanning speed, and 1000 PPI. A 198 µm channel depth was measured for the channel filled uniformly with 100% black color (RGB: 0) (Figure 6a). For the channel filled with 80% black (RGB: 51), the channel depth was measured to be 177 µm (Figure 6b). The width of the fabricated channels was calculated for dimensions repeatability check, and an uncertainty of ±6 µm was calculated. The exact process was repeated for different operating conditions. For the current application, a channel depth of approximately 110 µm was measured for a 100% black channel fabricated at 21 watt laser power (70%), 250 mm/s scanning speed (100%), and 1000 PPI. For the same conditions, around 70 µm channel depth was observed for a 40% black (RGB: 153) channel. Therefore, a gradual fill starting with 100% black to 40% black was used to obtain the required slope of the trapezoidal cross-section microfluidic channel (110/70 µm).

**Figure 7:**
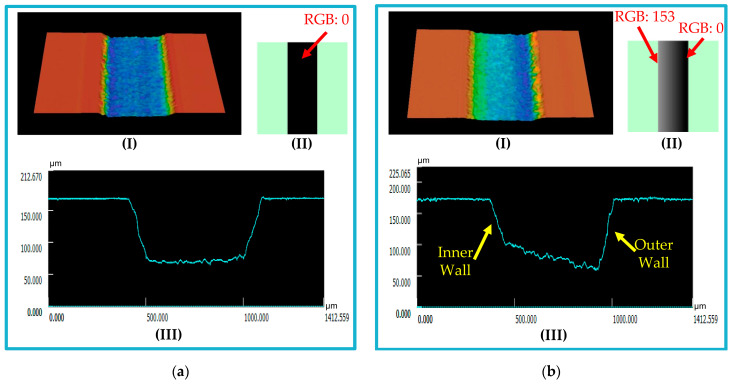
(**a**) Black (RGB: 0) uniform fill is applied in a microfluidic channel design for a rectangular cross-section channel. (**b**) Gradual fill, from black (RGB: 0) to 40% black (RGB: 153), is used for a trapezoidal cross section. (I) 3D channel image, (II) the applied fill in the design of the channel, and (III) the channel profile.

A surface roughness of 1.94 µm was obtained for the engraved surface. To enhance the surface roughness, the machined surface is first cleaned with DI water to remove any sticking dust. It is then exposed to a 200 °C hot air stream for 5 s. It can also be subjected to a direct flame from a 2 cm distance for 2 s as an alternative to hot air exposure. This short heat exposure time melts the unwanted small peaks or filaments created during fabrication without affecting the chip dimensions. After surface cleaning and processing, the surface roughness was measured to be 1.07 µm. Figure 7 presents fabricated microfluidic channels with their profile after surface cleaning and processing. Figure 7a shows a uniformly filled channel, while a gradually filled channel is presented in Figure 7b.

The chip was subjected to 150 °C for 15 min in the natural ventilation lab oven to study the thermal effect of bonding temperature on the stability of channel dimensions. As there was no applied force on the surface of the chip, and the set temperature was lower than the melting temperature of PMMA, no chip dimensions change was measured. Chip deformations were noted at the same temperature with high bonding forces, and some changes in dimensions were measured. On the other hand, air bubbles were observed between the two PMMA layers when applying low forces. In our application, the optimal bonding force was found to be 2 N. As a result, the bonding conditions for 2 mm PMMA sheets were concluded to be at 150 °C under 2 N force for 15 min in a natural ventilation lab oven. Applying these conditions, a transparent chip without any deformations can be obtained, as noted in the chip revealed in Figure 4.

DI water was pumped into the fabricated chip with different flow rates for leakage testing, starting with 500 µL/min. The flow rate was increased using a 500 µL/min step until the inlet tube was released from the syringe output port at a 4000 µL/min flow rate. This release happened due to the back pressure created inside the channel at high flow rates and because there was no leakage between the two bonded PMMA layers. The application of high operating flow rates without leakage is considered one of the essential advantages of using PMMA for microfluidic systems instead of PDMS.

### 3.2. WBCs Separation

**Figure 8:**
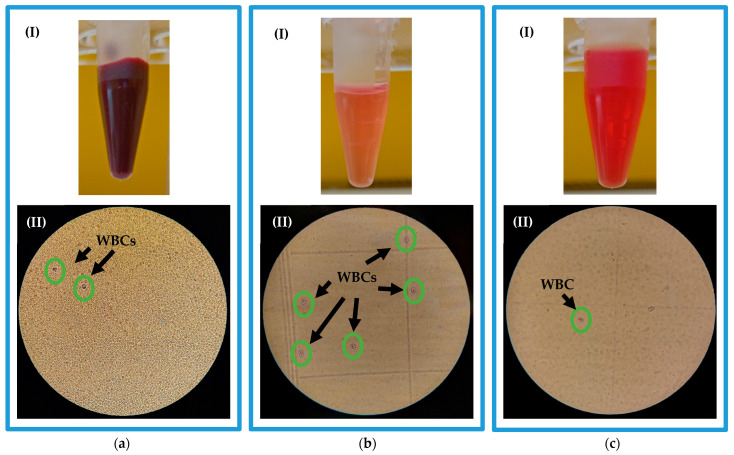
Blood samples (I) and hemocytometer microscopic images (II) of (**a**) a whole blood sample, (**b**) a collected sample from inner outlet, and (**c**) a collected sample from outer outlet.

Figure 8a displays a fresh whole blood sample intended for dilution and subsequent pumping into the microfluidic chip for WBCs separation. Gentian violet stain was utilized to aid in WBCs visualization due to their transparent nature. The hemocytometer microscopic image of the stained sample of Figure 8a reveals a small number of WBCs amidst a pool of RBCs. As a result, the high concentration of RBCs causes the lines of the hemocytometer counting area to vanish, rendering the counting process impossible for whole blood samples using this method. Therefore, dilution is necessary for accurate counting and to enhance the separation efficiency of the microfluidic chip by reducing cell-to-cell interaction. In the WBC separation process, the unstained diluted blood sample is injected into the chip, and the separated samples are collected from outlets. The collected samples are then stained for WBCs visualization. Figure 8b,c illustrates the samples collected from the inner and outer outlets, respectively. The light color of the inner outlet sample stems from a low RBC concentration, whereas the higher RBC concentration in the outer outlet results in a darker sample color. Figure 8b showcases numerous WBCs against a clear background, while Figure 8c exhibits a single WBC surrounded by a turbid background. The increased turbidity in the outer outlet sample is attributed to the remnants of lysed RBCs.

**Figure 9:**
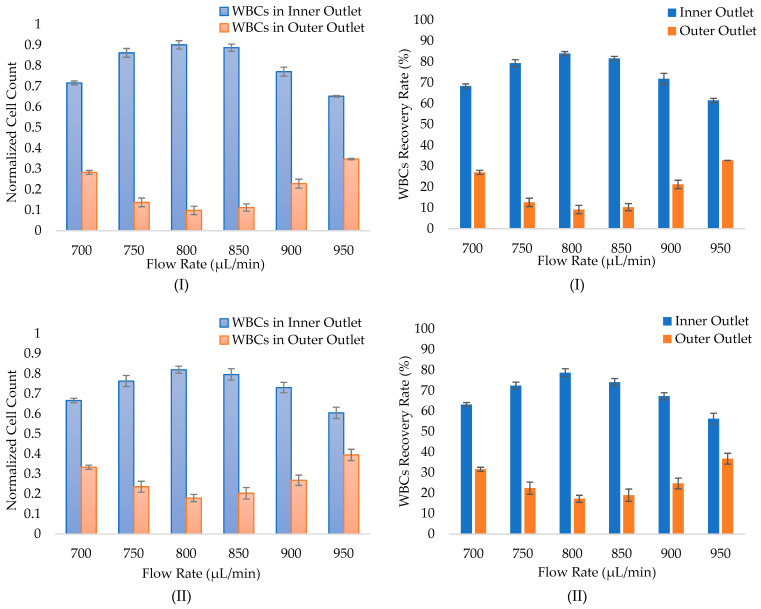

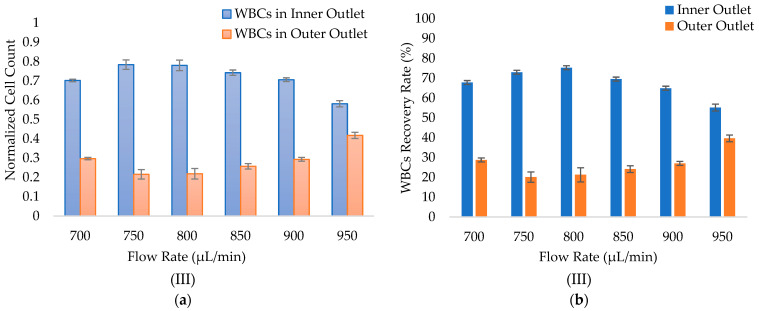
Hemocytometer WBCs count in the inner and outer outlets (**a**), and WBCs recovery rate in the inner and outer outlets (**b**) at different flow rates for 1% (I), 2% (II), and 3% (III) hematocrit blood samples.

The WBCs separation experiments were conducted firstly at 500, 1000, 1500, and 2000 µL/min flow rates to define the process operating flow rate range. All experiments were first performed on 1% hematocrit-diluted blood samples. Approximately 24, 36, 13, and 6% of separation efficiencies were observed for 500, 1000, 1500, and 2000 µL/min flow rates, respectively. Therefore, the same methods and experiments were applied at flow rates from 600 to 1000 µL/min with a 50 µL/min increment to define the optimal flow rate. For studying the effect of dilution factor of the sample and for further verification of the functionality of the fabricated chip, the separation procedure has been repeated for 2% and 3% hematocrit diluted blood samples. Figure 9a demonstrates a normalized cell count of WBCs in the inner and outer outlets of the fabricated spiral microfluidic chip for 1%, 2%, and 3% hematocrit samples. It has been noted that at low flow rates, WBCs were randomly distributed in both spiral channel outlets. Increasing the sample pumping flow rate increases the number of WBCs in the inner outlet of the channel. With further flow rate increases, a randomly distributed cell behavior occurs, and the number of WBCs in the inner outlet is reduced again. This complies with the fact that the inertial lift forces for the 12–15 µm WBCs are dominant to the Dean drag forces at higher flow rates [48,49].

WBCs recovery rate in the inner and outer outlets for 1%, 2%, and 3% hematocrit samples are shown in Figure 9b. Results reveal that the fabricated chip is capable of separating WBCs from whole blood with the highest recovery rate in the inner outlet for the three diluted blood samples at 800 µL/min flow rate. It can also be clearly noted from Figure 9b that the WBCs recovery rate in the inner outlet for the same flow rate increases with the increase in the dilution factor. The recovery rate of WBCs in the inner outlet peaks at 84% for 1% hematocrit sample. This aligns with the findings reported in the literature [26,33], which indicate recovery rates of CTCs ranging from 82% to 89%. 

**Figure 10:**
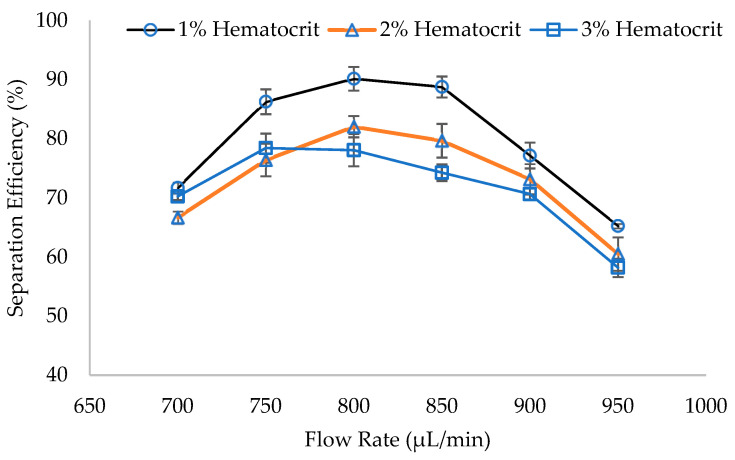
WBCs separation efficiency at different flow rates of 1%, 2%, and 3% hematocrit blood samples.

The highest separation efficiencies calculated from experiments conducted at different flow rates are shown in Figure 10. The optimal flow rate was observed to be 800 µL/min for 1% and 2% hematocrit samples, while WBCs separation efficiency of 3% hematocrit sample peaks at 750 µL/min. A WBCs separation efficiency of 90.14% was recorded for 1% hematocrit diluted blood samples, compared to separation efficiencies of 82.03% and 78.42% for 2% and 3% hematocrit samples. Consistent with previous findings [7], a further increase in dilution factor, which may reach 700×, increases the separation efficiency. The decrease in the separation efficiency with the increase in hematocrit is due to the higher concentration of RBCs in higher blood hematocrit samples. This heightened RBC concentration leads to intensified cell-to-cell interactions, resulting in a degradation of the separation efficiency. Through these experiments, the ability of the fabricated microfluidic chip using the proposed methodology for cell separation has been demonstrated.

Because the main contribution of the present work is concentrated on the fabrication of the chip, dilution factors of 51× (1% hematocrit), 22.5× (2% hematocrit), and 17× (3% hematocrit) were only used in the WBCs separation process for verification. Further enhancement in separation efficiency can also be conducted by optimization of the channel dimensions, the trapezoidal slope, and the radius of spiral curvature. The optimal flow rate for cell separation differs based on the fabricated channel dimensions and trapezoidal slope. That happens because of the changes in pressure profile across the channel, inertial forces, and Dean drag forces. As a result, the final equilibrium positions of particles will be changed. The enhancement of surface roughness of engraved surfaces using a CO_2_ laser beam on PMMA will be a subject of more profound studies, and some experiments using a laser lens with a smaller focal spot will be performed in the future. This enhancement may also increase the separation efficiency.

Using the method presented in this article reduces the cost and time of fabrication of microfluidic channels in general and trapezoidal cross-section channels in particular. In addition to the low cost and availability of PMMA material, there is no need for etching chemicals, clean room environment, or expensive equipment to help achieve this reduction in cost and fabrication time. The fabrication of a chip costs less than 30 cents, and the total fabrication time is 20 min. The fabrication time includes laser engraving, surface cleaning and processing, and thermal bonding. Table 1 illustrates a comparison between the present work and previous methods for the fabrication of trapezoidal cross-sectional spiral microfluidic channels.

## 4. Conclusions

In the current work, a trapezoidal cross-section spiral microfluidic chip for particle separation was fabricated with a meagre cost (30 cents) and low fabrication time (20 min). The low fabrication time and cost make the chip suitable for disposable single-use biomedical applications. The fabrication was performed using direct engraving of a CO_2_ laser beam on PMMA material. A grayscale approach was used to solve complex fabrication problems of trapezoidal cross-section microfluidic channels. Using grayscale in design controlled the channel depth and slope. The low bonding temperature (150 °C) used in this work allowed the implementation of other components, such as sensors, with the microfluidic channels on the same chip. Experimental tests showed that the fabricated chip could withstand high operating flow rates without leakage or channel deformation. The capability of the presented fabrication technique to be applied in microfluidic applications was demonstrated by WBC separation from whole blood experiments at different dilution factors. The experimental results reported a separation efficiency of 90.14% for a 1% hematocrit diluted blood sample using the fabricated chip, with an optimal flow rate of 800 µL/min. In addition to WBC separation, the developed chip is beneficial to other cell-sorting applications, including the isolation of intracellular microorganisms like brucellae from blood cells.

## Figures and Tables

**Table 1:** Comparison between the fabrication of trapezoidal cross-sectional spiral channels using the present method and some previous methods.

Reference	[1]	[24]	The Present Work
**Material**	Glass	PDMS	PMMA
**Fabrication technology**	Femtosecond laser	Soft lithography	CO_2_ laser
**Operating flow rate**	High	Low	High
**Surface Roughness**	0.27 µm	0.8 µm	1.07 µm
**Bonding**	Thermally at 620 °C in a muffle furnace	Oxygen plasma treatment	Thermally at 150 °C in a natural ventilation oven
**Fabrication time**	7 h and 9 min	~1 day	20 min
**Estimated cost/chip**	>$5	>$5	<30 cents

## Data Availability

No new data were created or analyzed in this study. Data sharing is not applicable to this article.
